# Upregulation of the *Nr2f1*-*A830082K12Rik* gene pair in murine neural crest cells results in a complex phenotype reminiscent of Waardenburg syndrome type 4

**DOI:** 10.1242/dmm.026773

**Published:** 2016-11-01

**Authors:** Karl-F. Bergeron, Chloé M. A. Nguyen, Tatiana Cardinal, Baptiste Charrier, David W. Silversides, Nicolas Pilon

**Affiliations:** 1 Molecular Genetics of Development Laboratory, Department of Biological Sciences andBioMed Research Center, University of Quebec at Montreal (UQAM), Montreal, H2X 3Y7, Canada; 2 Veterinary Genetics Laboratory, Faculty of Veterinary Medicine, University of Montreal, St-Hyacinthe, J2S 7C6, Canada

**Keywords:** Enteric nervous system, Hirschsprung disease, Melanocytes, Mouse model, Neural crest cells, Waardenburg syndrome

## Abstract

Waardenburg syndrome is a neurocristopathy characterized by a combination of skin and hair depigmentation, and inner ear defects. In the type 4 form, these defects show comorbidity with Hirschsprung disease, a disorder marked by an absence of neural ganglia in the distal colon, triggering functional intestinal obstruction. Here, we report that the *Spot* mouse line – obtained through an insertional mutagenesis screen for genes involved in neural crest cell (NCC) development – is a model for Waardenburg syndrome type 4. We found that the *Spot* insertional mutation causes overexpression of an overlapping gene pair composed of the transcription-factor-encoding *Nr2f1* and the antisense long non-coding RNA *A830082K12Rik* in NCCs through a mechanism involving relief of repression of these genes. Consistent with the previously described role of Nr2f1 in promoting gliogenesis in the central nervous system, we further found that NCC-derived progenitors of the enteric nervous system fail to fully colonize *Spot* embryonic guts owing to their premature differentiation in glial cells. Taken together, our data thus identify silencer elements of the *Nr2f1*-*A830082K12Rik* gene pair as new candidate loci for Waardenburg syndrome type 4.

## Abstract (precis)

**Summary:** A forward genetic screen approach unveils a new mechanism, involving *Nr2f1* and/or *A830082K12Rik* overexpression in neural crest cells, that could promote development of Waardenburg syndrome and Hirschsprung disease.

## INTRODUCTION

Neural crest cells (NCCs) form a vertebrate-specific population of multipotent stem cells that is induced at the border between the neural and non-neural ectoderm soon after initiation of gastrulation (Stuhlmiller and Garcia-Castro, 2012). During neurulation, the edges of the neural plate move upwards and then fuse, placing the recently induced NCCs in the dorsal-most portion of the closing neural tube. NCCs subsequently undergo a wave of delamination and migration, beginning at the anterior part of the neural tube and moving posteriorly. This results in the colonization of a wide variety of embryonic structures to which NCCs contribute diverse cell types, such as melanocytes (pigment cells) as well as peripheral neurons and glia (Bronner and LeDouarin, 2012). Abnormal NCC development is a root cause of numerous birth defects (e.g. aganglionic megacolon, cleft lip and palate, and persistent truncus-arteriosus) and cancers (e.g. melanoma and neuroblastoma), which are collectively known as neurocristopathies (Bolande, 1997). Because of the wide array of NCC derivatives, distinct structures and cell types (isolated or in combination) are affected in each of these pathologies. The underlying genetic and molecular causes of most neurocristopathies are poorly understood (Etchevers et al., 2006).

The best-known neurocristopathy is Hirschsprung disease (HSCR), which is also commonly named aganglionic megacolon. HSCR is a severe congenital malformation of the enteric nervous system (ENS) that is found in 1 of every 5000 newborn infants and that affects males more frequently than it does females (Amiel et al., 2008). This life-threatening condition is caused by the incapacity of NCC-derived enteric neural progenitors to reach the end of the bowel during development (Bergeron et al., 2013; Goldstein et al., 2013; Lake and Heuckeroth, 2013). In the absence of such progenitors, the affected segment is devoid of peristalsis-controlling neural ganglia, resulting in tonic muscle contraction and functional bowel obstruction. The vast majority of enteric neural progenitors are specifically derived from NCCs originating from the vagal region of the neural tube. The particular rostrocaudal migration pathway adopted by such ‘enteric’ NCCs (ENCCs) within the developing intestines is thought to be one of the main contributing factors to HSCR pathogenesis. In the mouse, these cells initially invade the foregut mesenchyme around embryonic day (E)9.5, reaching the end of the hindgut by E14.5. It is also important to note that completion of ENS formation is not possible without tight coordination of ENCC migration with cell proliferation, survival and differentiation mechanisms. Defects in any of these processes can thus be a source of aganglionosis in terminal intestinal segments (Obermayr et al., 2013).

Although HSCR occurs most commonly as an isolated trait, it can also occur as part of a syndrome involving other NCC defects (Amiel et al., 2008; Bergeron et al., 2013). One such syndrome is Waardenburg syndrome type 4 (also known as Waardenburg–Shah syndrome) that combines aganglionic megacolon, depigmentation of skin and hair, as well as inner ear defects that could affect hearing and/or balance (Pingault et al., 2010; Tachibana et al., 2003). Depigmentation and inner ear defects are defining features of all four sub-types of Waardenburg syndrome, and are both due to a lack of NCC-derived melanocytes (Pingault et al., 2010). Although hearing loss is generally considered to be a cardinal feature of Waardenburg syndrome, it is noteworthy that both its prevalence (25-88% of cases depending of sub-type) and severity (unilateral or bilateral, mostly progressive) are highly variable (Black et al., 2001; Pingault et al., 2010). This contrasts with the fact that signs of balance-related problems (dizziness, vertigo or disequilibrium), to which much less attention has been given in the literature, could be present in as high as 75-90% of cases (Black et al., 2001; Hageman and Oosterveld, 1977; Hildesheimer et al., 1989). Loss-of-function mutations in six genes (including both coding and regulatory sequences) have been associated with the different types of Waardenburg syndrome. Four of these genes encode a transcription factor and two others encode a ligand-receptor pair: *PAX3* (type 1 and 3), *MITF* (type 2), *SNAI2* (type 2), *SOX10* (type 2 and 4), and *EDN3* and *EDNRB* (ligand and receptor, type 4). Although most cases of Waardenburg syndrome type 1 and 3 can be explained by mutations of *PAX3*, it is estimated that up to 70% of type 2 and 35% of type 4 cases cannot currently be explained by mutation of a known gene (Pingault et al., 2010).

The inner ear is a sophisticated organ, comprising several interconnected fluid-filled ducts and chambers that are arranged in two main compartments named the vestibule and the cochlea (Forge and Wright, 2002; Groves and Fekete, 2012). The vestibule contains the sensory organs (utricle, saccule and ampullae) controlling balance, whereas the snail-shaped cochlea contains the sensory organ (organ of Corti) that controls hearing. These sensory organs are characterized by the presence of mechanosensory hair cells in association with supporting cells. The mechanosensory hair cells are innervated through the cochleovestibular nerve (cranial nerve VIII), and they detect sounds and movements through stereocilia immersed in a fluid called endolymph. The mechanotransduction function of these sensory cells is crucially dependent on the unusual K^+^-enriched ionic composition of endolymph, on which cochlear melanocytes (also called intermediate cells) of the stria vascularis are known to play a key role (Tachibana, 1999; Zdebik et al., 2009). Although the similar distribution of melanocytes around the utricle and ampullae has been described a long time ago in both human and rodents (Escobar et al., 1995; LaFerriere et al., 1974; Masuda et al., 1995), the function of such vestibular melanocytes remains currently unclear (Barozzi et al., 2015; Masuda et al., 2001; Sanchez Hanke et al., 2005).

Here, we report the generation and characterization of a novel mouse model for Waardenburg syndrome type 4, which involves overexpression of the transcription-factor-encoding gene *Nr2f1* and its overlapping antisense long noncoding (lnc)RNA *A830082K12Rik* in NCCs. In addition to providing the first phenotypic confirmation that premature gliogenesis can be a cause of aganglionic megacolon in postnatal mice, our work also points to a key role for vestibular melanocytes in regulating endolymph homeostasis.

## RESULTS

### Phenotypic overview of the *Spot* mouse line

The *Spot* mouse line was generated through a forward genetic screen for new loci important for NCC development. This screen was based on random integration of a tyrosinase minigene in the genome of FVB/N albino mice, using rescue of pigmentation as a marker for transgenesis as well as for NCC anomalies. Most mice obtained in this way are uniformly pigmented, ranging from light gray to dark brown (Methot et al., 1995). However, a few mutants display pigmentation abnormalities, suggesting the insertion has disrupted a genetic locus important for melanocyte and, by extension, NCC development (Pilon, 2016). Other details about this screen, including the detailed description of two other lines that arose from it (*TashT* and *Holstein*) can be found elsewhere (Bergeron et al., 2015; Soret et al., 2015).

**Fig. 1.**
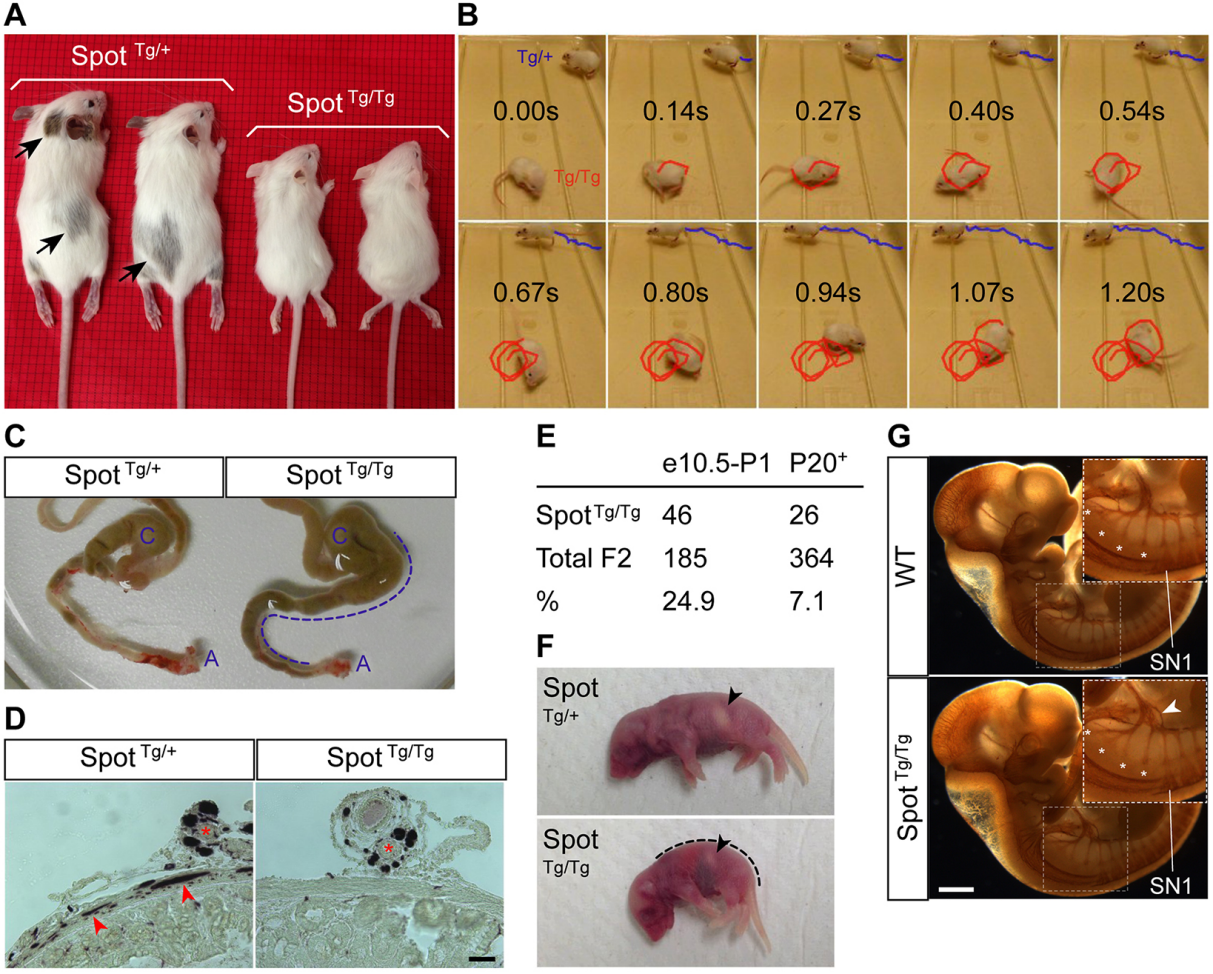
***Spot* mice display pigmentation, balance and peripheral innervation defects.** (A) P20 *Spot^Tg/Tg^*;G4-RFP mice (*Spot*^Tg/Tg^) display allele dosage-dependent depigmentation. Spotted areas of pigmentation (arrows) are only present in heterozygotes. (B) Time-lapse series (time indicated in seconds) of a P20 *Spot*^Tg/Tg^ animal exhibiting spinning behavior (red path). A control *Spot*^Tg/+^ sibling was seen walking normally in the background (blue path). (C) Dissected intestines of P20 *Spot*^Tg/Tg^ animals displayed classic signs of megacolon, including blockage within the distal colon and upstream accumulation of fecal material (dotted line). C, cecum; A, anus. (D) Immunohistochemistry analysis for neuronal βIII-tubulin on 10-µm transverse sections of P20 *Spot* distal colons. Arrowheads outline myenteric neural ganglia, whereas asterisks label clusters of extrinsic nerve fibers associated with mesenteric veins. (E) Frequency of homozygotes amongst *Spot* F2 animals during development (between E10.5 and P1) versus at weaning age (P20+). (F) *Spot*^Tg/Tg^ neonates often display dropping forelimbs, abnormal curvature (dotted line) and a smaller milk spot (arrowheads). (G) Whole-mount staining of peripheral nerves in E10.5 embryos using neurofilament (2H3) immunohistochemistry. For a majority of *Spot*^Tg/Tg^ embryos (5 out of 7), the most anterior spinal nerve (SN1) was improperly connected to the hypoglossal nerve (arrowheads) in a unilateral manner. Asterisks indicate the position of hypoglossal nerve roots exiting the ventral neural tube. WT, wild-type FVB embryo. Scale bars: 50 µm (D); 500 µm (G).

The *Spot* line got its name because of the presence of a few large spots of light gray pigmentation in heterozygotes (*Spot*^Tg/+^). Of note, these spots appeared darker following backcrossing of the *Spot* allele onto a Gata4p[5kb]-GFP (G4-GFP) or G4-RFP transgenic background (Fig. 1A) – both of which conveniently confer endogenous fluorescent labeling to migratory NCCs (Pilon et al., 2008). Irrespective of genetic background (wild-type FVB, G4-GFP or G4-RFP), homozygous *Spot* mice (*Spot*^Tg/Tg^) displayed additional fully penetrant phenotypes at weaning age (>25 cases observed), including complete lack of hair-coat pigmentation (Fig. 1A), hyperactive circling (or ‘spinning’) (Fig. 1B and Movie 1) and hallmarks of aganglionic megacolon in rodents (retarded growth, lethargy, hunched posture, distended abdomen and premature death). Aganglionic megacolon was confirmed by observing an accumulation of fecal material in the proximal colon and cecum due to blockage in the distal colon (Fig. 1C), combined with a lack of myenteric ganglia in this region (Fig. 1D). No sex bias was observed for any of these phenotypes.

### Perinatal death of a majority of *Spot*^Tg/Tg^ animals

Genotyping of F2 litters showed that only about 7% of *Spot* animals were homozygous at weaning. However, the expected mendelian ratio of *Spot*^Tg/Tg^ animals was found to be present in prenatal and newborn litters (Fig. 1E), suggesting that many homozygotes die within a few days after birth. From birth onwards, *Spot* litters were systematically examined to identify the stage and the likely cause of death. This analysis revealed that the perinatal lethality of *Spot*^Tg/Tg^ animals occurs between postnatal day (P)1 and P2, with dying pups having less milk in their stomach than their surviving littermates (Fig. 1F). In the absence of overt anomalies affecting the cranio-facial region or the internal organs, these observations indicate that defective neural control of suckling and/or swallowing might be the cause of death. This was also strongly supported by the presence of other signs of neuromuscular defects in *Spot*^Tg/Tg^ neonates, such as dropping forelimbs and abnormal body curvature (Turgeon and Meloche, 2009). To verify this possibility, the status of cranio-facial innervation was investigated by immunostaining of embryos at E10.5 with the 2H3 anti-neurofilament antibody. Interestingly, examination of the cranial nerves involved in the control of suckling and/or swallowing (i.e. V, VII, IX, X and XII) revealed an abnormal connection between the spinal nerve I and the cranial nerve XII (hypoglossal) in 71% of the tested *Spot*^Tg/Tg^ embryos (Fig. 1G). This defect was found to be unilateral, always affecting the right side. Although we cannot currently explain the intermittent nor the unilateral nature of this neural defect, we believe that it could be a contributing factor to the early postnatal death of *Spot*^Tg/Tg^ animals.

### Lack of vestibular melanocytes and endolymph liquid underlies the balance problems of *Spot*^Tg/Tg^ mice

**Fig. 2.**
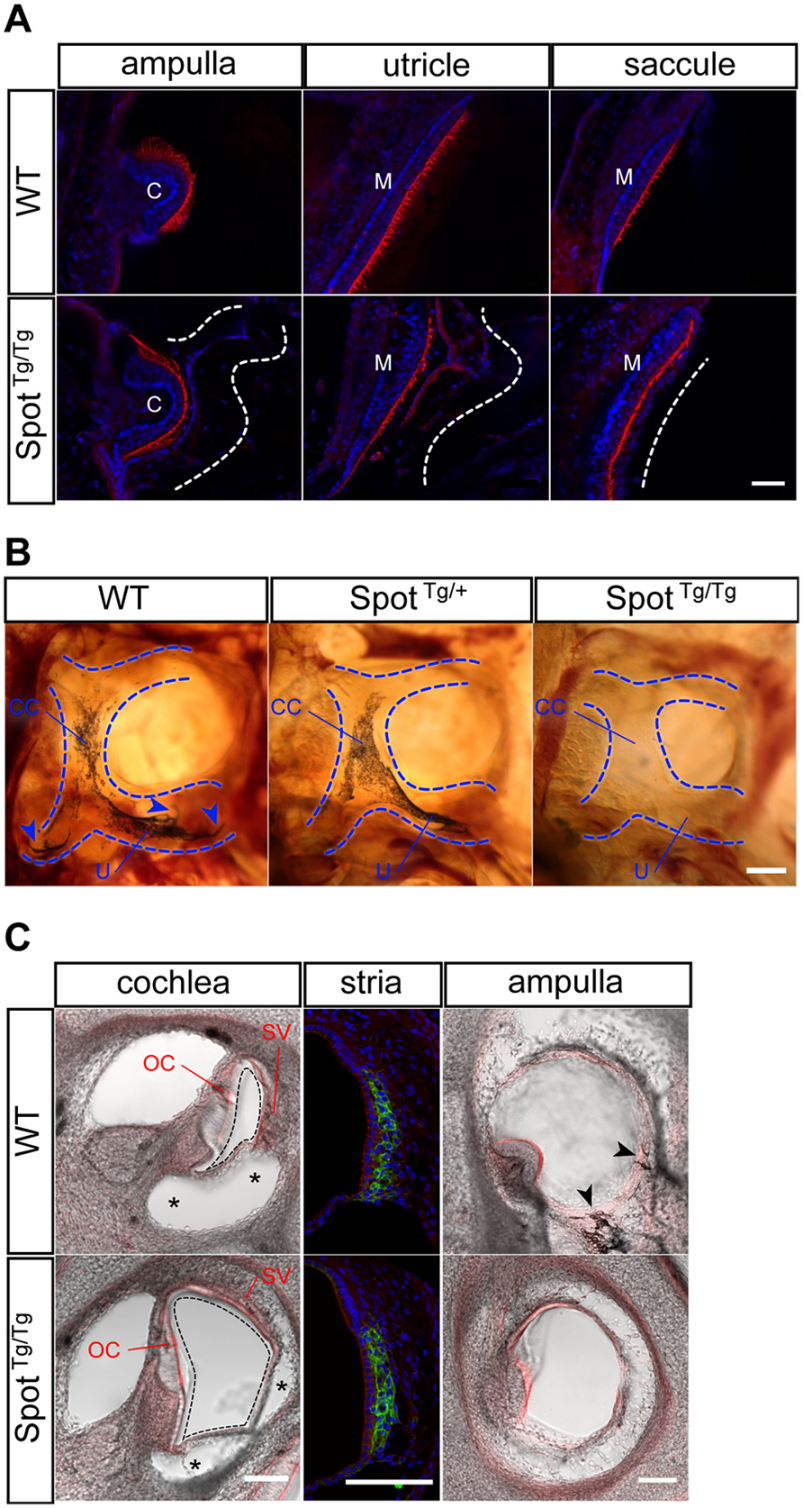
**Absence of endolymph liquid and melanocytes in the vestibule underlies the balance problems of *Spot*^Tg/Tg^ mice.** (A) Single confocal slices of vestibular sensory organs (ampulla, utricle and saccule) within inner ear sections (200 µm) of P20 mice [*Spot*^Tg/Tg^;G4-RFP (*Spot*^Tg/Tg^) versus G4-RFP (WT)] stained with DAPI (which labels cell nuclei in blue) and TRITC–Phalloidin (which labels actin filaments in red). The hair-cell-containing sensory epithelia (C, crista; M, macula) of *Spot*^Tg/Tg^ mice are covered with a collapsed cellular membrane (delineated by a dashed line). (B) Intracranial view of methylsalicylate-cleared P20 temporal bones from an allelic series of *Spot*;G4-RFP animals showing allele-dosage-dependent loss of melanocytes within the vestibular membranous labyrinth (outlined in blue) of *Spot* mice, including the three ampullae (arrowheads). CC, common crus; U, utricle. (C) Confocal imaging of sensory structures within sections (100 µm) of P1 inner ears [*Spot*^Tg/Tg^;G4-RFP (*Spot*^Tg/Tg^) versus G4-RFP (WT)] labeled with DAPI (which labels cell nuclei in blue), TRITC–Phalloidin (which labels actin filaments in red) and an anti-Kit antibody (which labels melanocytes in green). In *Spot*^Tg/Tg^ cochlea, the endolymph-containing scala media (black dashed line) had expanded at the expense of the perilymph-containing scala vestibuli (asterisks) (left panels) but Kit-positive melanocytes were normally detected in the stria vascularis (middle panels). In contrast, pigmented melanocytes that are normally found around ampullae were not detected in *Spot*^Tg/Tg^ animals (right panels). OC, organ of Corti; SV, stria vascularis. All panels are representative images of three animals per genotype. Scale bars: 50 µm (A); 250 µm (B); 100 µm (C).

Balance problems, such as the spinning behavior exhibited by *Spot*^Tg/Tg^ animals that survived beyond P2, are indicative of vestibular malfunction. To determine the etiology of this phenotype, we first analyzed the bony labyrinth of animals at P20 by performing micro-computed tomography, which failed to reveal differences between mutants and controls (Fig. S1). In contrast, analysis of the membranous labyrinth with confocal imaging revealed that *Spot*^Tg/Tg^ vestibules at P20 have a greatly reduced endolymphatic space, as evidenced by the collapse of the vestibular membrane on the varied sensory epithelia (Fig. 2A). This phenotype suggests defective endolymph production and/or homeostasis, processes for which cochlear melanocytes residing in the stria vascularis are crucially required (Tachibana et al., 2003; Zdebik et al., 2009). In agreement with such a role for vestibular melanocytes as well, further analyses revealed that pigment cells were almost completely absent in *Spot*^Tg/Tg^ vestibules (Fig. 2B). *Spot*^Tg/+^ vestibules displayed an intermediate phenotype, with melanocytes present in the common crus and utricle but virtually absent from all three ampullae (Fig. 2B). Confocal imaging of tissues in newborns not only confirmed the developmental origin of this defect but also its restriction to the vestibule. Indeed, no difference in the numbers of Kit^+^ melanocytes could be noted in the stria vascularis of *Spot*^Tg/Tg^ neonates (Fig. 2C). We also noticed that, although mutant cochleae had an expanded endolymphatic space, their associated organ of Corti was structurally intact (Fig. 2C and Fig. S2). Accordingly, *Spot*^Tg/Tg^ animals appeared to hear properly, as suggested by their normal behavior during the Preyer's reflex test at P21 (Movie 1) – the oldest age at which this test can be performed given the lethal megacolon phenotype.

### Premature glial differentiation is the cause of *Spot* ENS defects

**Fig. 3.**
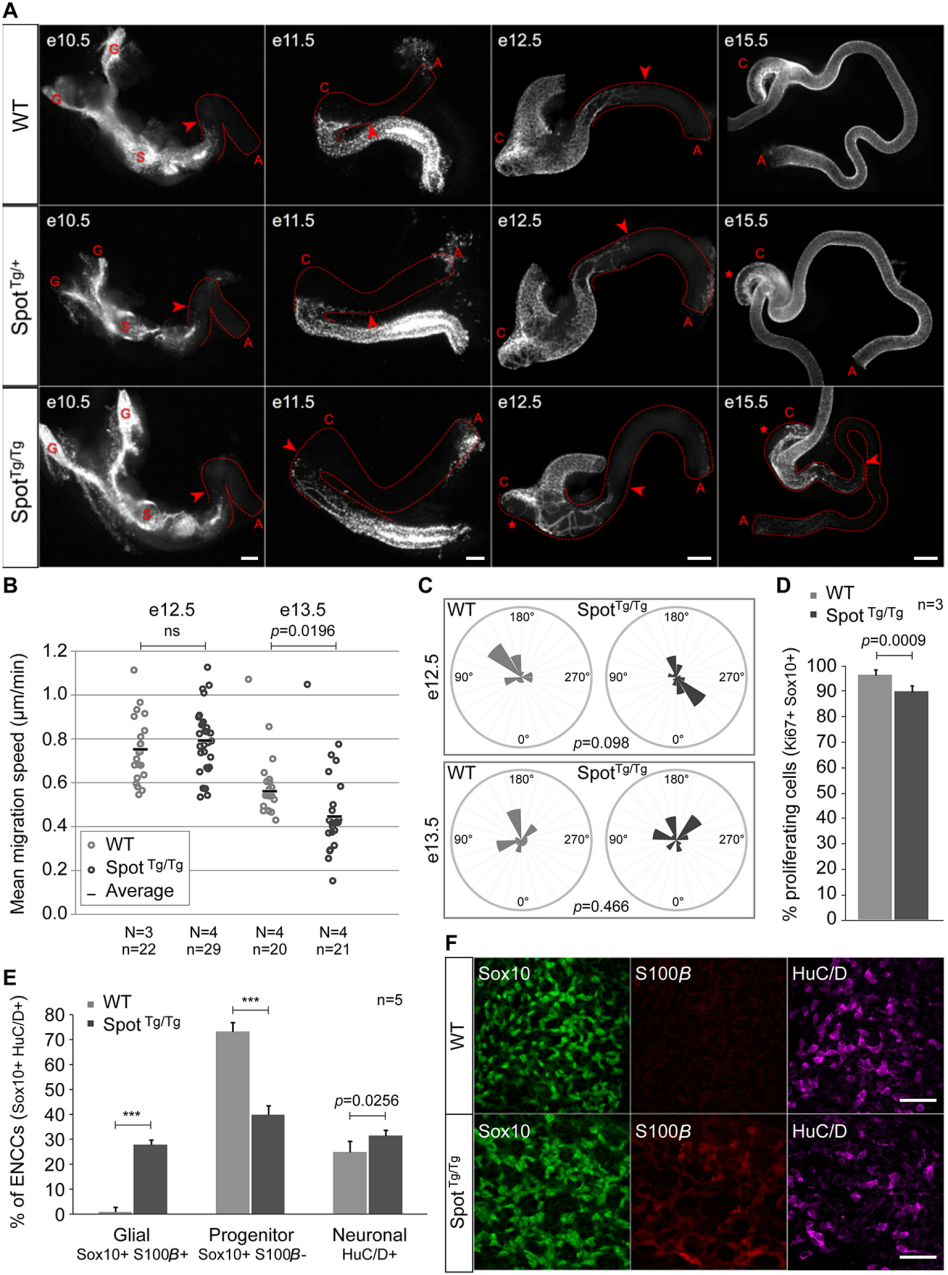
**Premature glial differentiation is the source of the ENS defect in *Spot* animals.** (A) Dissected embryonic intestines of an allelic series of *Spot*;G4-GFP animals at E10.5, E11.5, E12.5 and E15.5 (for each stage, *n*=4 per genotype). Where relevant, arrowheads point to the migration front. Asterisks indicate a lack of ENS progenitors in the cecum of *Spot*^Tg/+^ and *Spot*^Tg/Tg^ embryos relative to wild type (WT, G4-GFP control). G, vagal ganglion; S, stomach; C, cecum; A, anus. (B) Live imaging and quantification of ENCC movement in colons of *Spot*^Tg/Tg^;G4-RFP (*Spot*^Tg/Tg^) and G4-RFP (WT) embryos at E12.5 and E13.5. Mean speed was calculated from individual cells at the tip of migrating arms over a 6-h period. *N*, number of colons analyzed; *n*, number of cells tracked; *P*, Student's *t*-test *P*-value; ns, not significant. (C) Directionality of ENCC movement in colons of *Spot*^Tg/Tg^;G4-RFP (*Spot*^Tg/Tg^) and G4-RFP (WT) embryos (quantified in B) relative to the mesentery axis. Likelihood ratio test *P*-value for circular data is indicated. (D) Proliferation of Sox10-positive ENCCs in the proximal intestine of *Spot*^Tg/Tg^;G4-RFP (*Spot*^Tg/Tg^) and G4-RFP (WT) E12.5 embryos (*n*=3 per genotype; Student's *t*-test). (E) Quantification of undifferentiated progenitors (Sox10-positive S100β-negative), as well as glial-fated (Sox10-positive S100β-positive) and neuronal-fated (HuC/D-positive) cells, within the ENCC population in the proximal colon of *Spot*^Tg/Tg^;G4-RFP (*Spot*^Tg/Tg^) and G4-RFP (WT) E15.5 embryos (*n*=5 per genotype). ***P<0.0001 (Student's *t*-test). (F) Confocal projections of the developing ENS (combining Sox10-positive and HuC/D-positive cells) within the proximal small intestine of *Spot*^Tg/Tg^;G4-RFP (*Spot*^Tg/Tg^) and G4-RFP (WT) E13.5 embryos, showing the precocious appearance of the glial marker S100β in Sox10-positive cells of *Spot*^Tg/Tg^ embryos (*n*=3 per genotype). Scale bars: 200 µm (A, E10.5–E12.5); 500 µm (A, E15.5); 50 µm (F).

To determine the origin of the aganglionic megacolon phenotype of *Spot*^Tg/Tg^ mutants, the extent of intestinal colonization by NCC-derived ENS progenitors was monitored during embryonic development. To this end, we took advantage of the fluorescent labeling provided by the G4-GFP transgene and found that a delay in ENCC colonization could be observed in *Spot*^Tg/Tg^ intestines starting at E11.5 (Fig. 3A). This defect was found to affect ENCCs passing through the cecum as well as those taking the trans-mesenteric route (Fig. S3) and to persist after the end of the normal period of colonization (i.e. E14.5) without signs of recovery at E15.5 (Fig. 3A). Quantification of the extent of colonization in *Spot*^Tg/Tg^ newborns by staining of acetylcholinesterase activity revealed that only about a quarter of the prospective colon was colonized in these animals, without significant differences between males and females (Fig. S4). Moreover, an additional permanent ENS formation defect that was characterized by reduced density in the cecum was observed in *Spot*^Tg/+^ embryos and was found to be exaggerated in *Spot*^Tg/Tg^ animals (Fig. 3A and Fig. S5).

Among the multiple neural crest derivatives that are affected in *Spot* animals, the developing ENS offers an ideal system for more comprehensive studies. Indeed, in addition to the fact that the basic principles of ENS formation are well known, embryonic gut tissues are relatively easy to access and well suited for *ex vivo* assays. We thus undertook a detailed characterization of the colonization defect described above, beginning with a comparison of speed and directionality of control (G4-RFP) and mutant (*Spot*^Tg/Tg^;G4-RFP) ENCCs in *ex vivo* cultures of whole embryonic intestines. Interestingly, each of these migration parameters was found to be affected in mutant ENCCs, but not in a concomitant manner. Indeed, although normal speed but aberrant directionality (albeit without reaching statistical significance) was detected at E12.5, a ∼30% decrease in mean migration speed and normal directionality was observed at E13.5 (Fig. 3B,C and Movie 2). An evaluation of the cellular processes affecting cell number further showed that proliferation (∼7% decrease), but not TUNEL^+^ cell death, was affected in *Spot*^Tg/Tg^ ENCCs at E12.5 (Fig. 3D and Fig. S6). These analyses were then complemented by an assessment of neuronal and glial differentiation. The use of a relatively late glial marker (S100β) for this third set of experiments allowed us to identify a very strong premature differentiation of *Spot*^Tg/Tg^ ENCCs towards the glial lineage in proximal colon at E15.5 (Fig. 3E). This striking increase in the number of glial-fated ENCCs (from 1 to ∼39% of total ENCCs in wild-type and mutant animals, respectively) was found to mainly occur at the expense of undifferentiated progenitors (Fig. 3E), an outcome also confirmed at an earlier stage in proximal small intestines at E13.5 (Fig. 3F). Given that premature differentiation (either neuronal or glial) has been previously reported to negatively impact ENCC migration and/or proliferation (Liu et al., 2015; Ngan et al., 2011; Okamura and Saga, 2008), we thus conclude from these experiments that premature gliogenesis is the primary cause of the *Spot* ENS defects.

### The *Spot* transgene insertion site is localized in non-coding sequences of the *Nr2f1*-*A830082K12Rik* overlapping gene pair

**Fig. 4.**
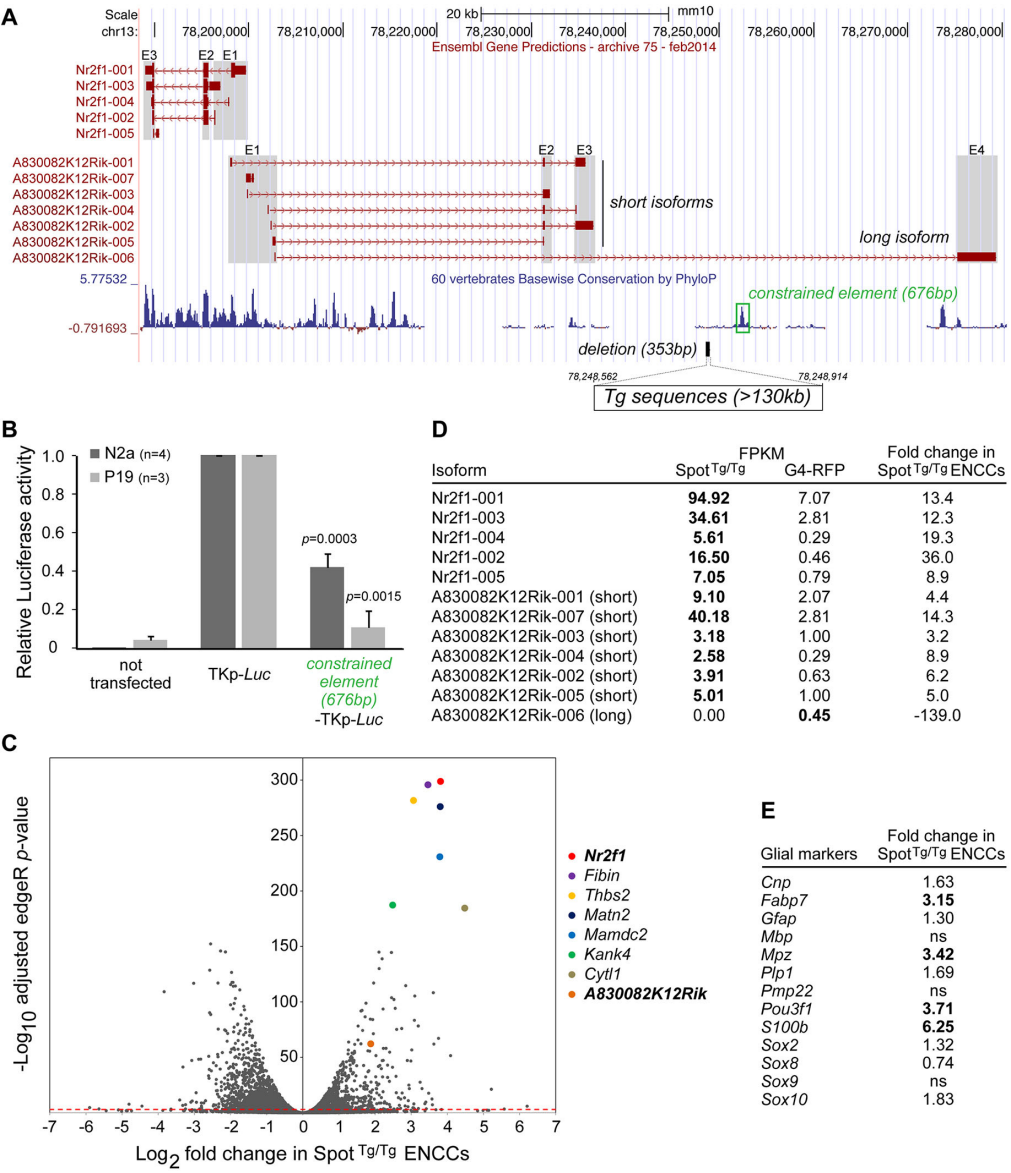
**Upregulation of the *Nr2f1*-*A830082K12Rik* gene pair in *Spot*^Tg/Tg^ ENCCs.** (A) Genomic location of the *Spot* transgene (Tg) insertion site on chromosome (Chr)13 within the last intron of the lncRNA gene *A830082K12Rik*, which is partly the antisense sequence of *Nr2f1*. Known isoforms of these two genes are presented, including their exon organization (E1, E2, E3 and E4; outlined in gray). Note the existence of short and long isoforms of *A830082K12Rik*. A 676-bp constrained element (PhyloP 60 vertebrates basewise conservation track; position 78,252,030-78,252,705; green box) is present next to a 353-bp deletion (black bar) induced by the transgene insertion. (B) Transcriptional activity of the 676-bp constrained element (green box in A) next to the *Spot* transgene insertion site was assessed in Neuro-2a (N2a) and P19 cells using a thymidine kinase minimal promoter (TKp) driving the expression of a luciferase (Luc) reporter [*n*=4 (N2a) or *n*=3 (P19) experiments; *P*, Student's *t*-test compared to TKp-*Luc* without constrained element]. (C) Volcano plot showing high-throughput differential expression data from E12.5 ENCCs [mutant *Spot*^Tg/Tg^;G4-RFP (*Spot*^Tg/Tg^) versus control G4-RFP]. The red dotted line represents the edgeR *P*-value cutoff (0.001), above which genes are considered to be statistically modulated in *Spot*^Tg/Tg^ ENCCs compared to control. (D) Modulation of *Nr2f1* and *A830082K12Rik* transcript prevalence in *Spot*^Tg/Tg^;G4-RFP ENCCs, as determined by performing Cufflinks analysis relative to control G4-RFP ENCCs. (E) Many glial markers were upregulated in E12.5 *Spot*^Tg/Tg^ ENCCs relative to control G4-RFP ENCCs. ns, not significant.

The *Spot* transgene insertion site was located by performing whole genome sequencing and subsequent analysis of mapped sequencing reads around the tyrosinase sequences, as per our previous work (Bergeron et al., 2015; Soret et al., 2015). Upon PCR amplification and Sanger sequencing of the boundaries between the transgene and genomic DNA, the insertion site was confirmed to be precisely located on chromosome 13 (cytoband C1) within the last intron of the uncharacterized gene *A830082K12Rik*, which is predicted to be expressed as multiple differentially spliced lncRNA species (Fig. 4A). Of note, this also places the insertion site in the vicinity of *Nr2f1* (also known as *Coup-tf1*; at ∼48.8 kb on the centromeric side), a gene encoding an orphan nuclear receptor notably known to promote gliogenesis in the central nervous system (Naka et al., 2008; Yamaguchi et al., 2004). From the mapping data, we further found that transgenesis resulted in a 353-bp deletion of genomic sequences as well as in the insertion of at least 130 kb of transgenic sequences. Other analyses of the sequences surrounding the *Spot* transgene insertion site revealed the presence of a peak of evolutionarily conserved non-coding sequences at ∼3.8 kb on the telomeric side of the insertion (Fig. 4A). This 676-bp region was cloned upstream of a minimal promoter driving expression of luciferase (TK109-*Luc*) and its regulatory potential was assayed in undifferentiated P19 embryocarcinoma and Neuro-2a neuroblastoma cells. A strong repressive transcriptional activity was observed in both cell types (Fig. 4B). As we previously observed for the *TashT* insertional mutant line (Bergeron et al., 2015), we concluded from these analyses that the transgenic insertion might have decoupled a long-range interaction between a constrained repressive element and the promoter of a surrounding gene.

### The *Nr2f1*-*A830082K12Rik* overlapping gene pair is upregulated in *Spot* ENCCs

To explore the consequences of the *Spot* transgenic insertion on both local and global gene expression, we compared the ribosomal (r)RNA-depleted transcriptome of control (G4-RFP) and mutant (*Spot*^Tg/Tg^;G4-RFP) ENCCs at E12.5, which were recovered by performing fluorescence-activated cell sorting (FACS). Given our previous detailed characterization of *Spot*^Tg/Tg^ ENCCs, this cell type was the logical choice for such an analysis. Our differential expression results indicated that thousands of genes (2955 genes at a *P*-value cut-off of 0.001) were significantly modulated in mutant ENCCs (Fig. 4C and Table S1). Although downregulated and upregulated genes were roughly equally distributed in this dataset, it is interesting to note that the most deregulated genes (in terms of both the fold change and the statistical significance) were all found in the upregulated group (Fig. 4C). Within this small subset, *Nr2f1* appeared to be the most significantly deregulated. Similarly, all short isoforms of *A830082K12Rik* (i.e. devoid of the last exon and thus uninterrupted by the transgenic insertion) were also found to be significantly upregulated in *Spot*^Tg/Tg^ ENCCs (Fig. 4D). By contrast, expression of the long isoform of *A830082K12Rik* (i.e. containing the last exon), which was normally weakly expressed in comparison to most short isoforms, was not detected in mutant ENCCs (Fig. 4D). Semi-quantitative reverse-transcriptase PCR (RT-PCR) validated our RNAseq results and additionally revealed that upregulation of *Nr2f1* and *A830082K12Rik* expression was not noticeable in whole embryonic intestines (Fig. S7), suggesting that these genes are specifically modulated in ENCCs. Perusal of our full transcriptome dataset further showed that none of the genes that have been previously implicated in Waardenburg syndrome type 4 were downregulated in 12.5 *Spot*^Tg/Tg^ ENCCs (Table S1). In fact, both *Ednrb* and *Sox10* were even found to be upregulated by 1.51- and 1.83-fold, respectively. In agreement with our finding of premature glial differentiation, we also noticed that several genes encoding glial markers were upregulated in *Spot*^Tg/Tg^ ENCCs at E12.5 (Fig. 4E and Table S1), especially *S100b* and *Mpz*, as well as the known Nr2f1 target genes *Fabp7* and *Pou3f1* (Montemayor et al., 2010; Yamaguchi et al., 2004). Gene ontology analysis of the significantly modulated gene set identified 243 significantly enriched terms (with ontology level ≥5 and *P*<0.05) that could be classified into eight main categories comprising (from most to least significant): nervous system development, cell differentiation and morphogenesis, metabolism, transport, cell division, cytoskeleton organization, cell adhesion, and response to stimuli (Fig. S8).

### Long-range repression of the *Nr2f1-A830082K12Rik* gene pair is disrupted in the *Spot* line

**Fig. 5.**
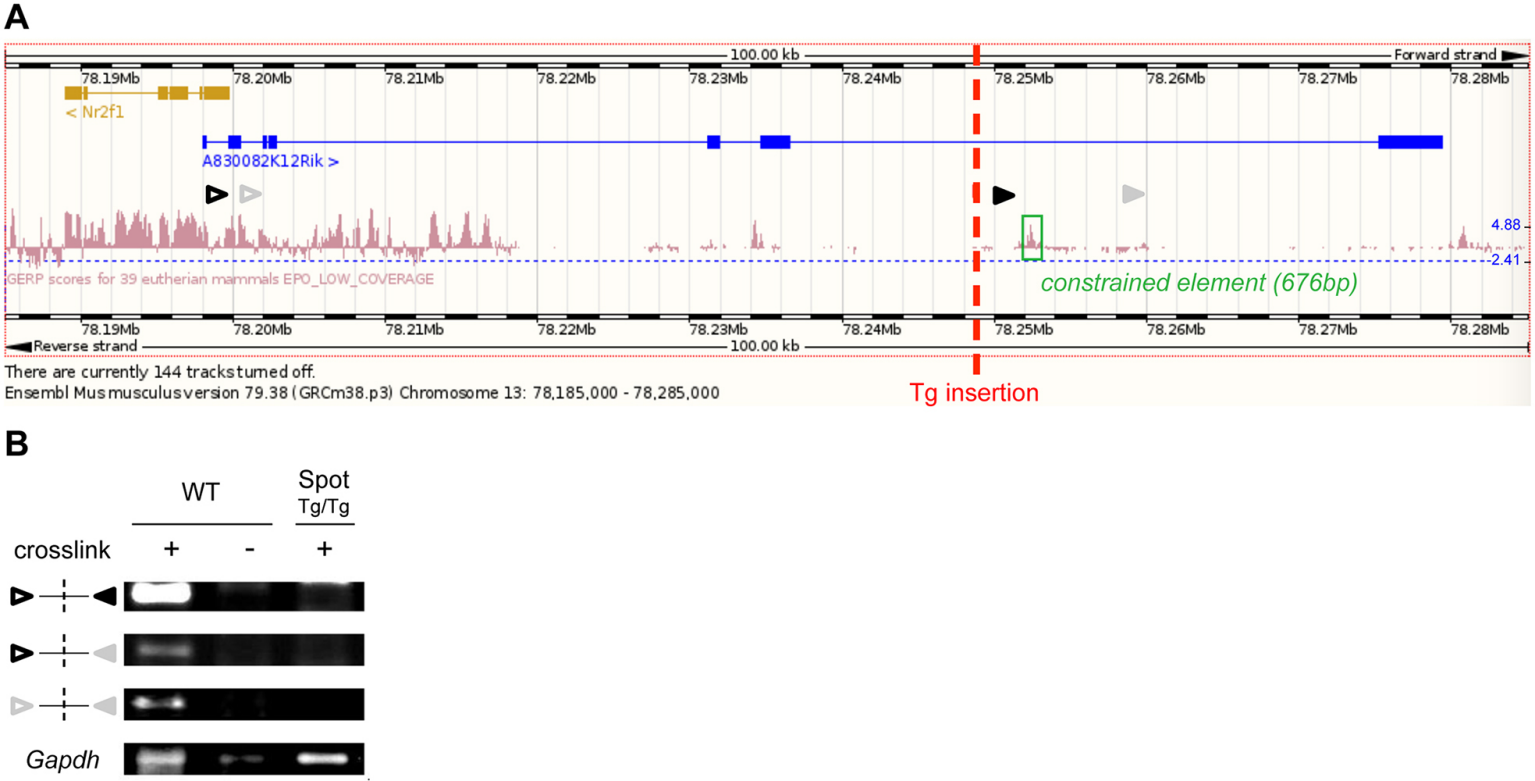
**Long-range repression of the *Nr2f1-A830082K12Rik* gene pair is disrupted in the *Spot* line.** (A,B) Evaluation of long-range interactions between the *Nr2f1-A830082K12Rik* gene pair and the 676-bp silencer element using chromosome conformation capture (3C) assays. The location of 3C primers (arrowheads, corresponding to PCR products shown in B) as well as that of the *Spot* transgene insertion site and the 676-bp constrained element are indicated in A. Results of PCR amplifications on 3C libraries made from wild-type (WT, control G4-RFP) and *Spot*^Tg/Tg^;G4-RFP (*Spot*^Tg/Tg^) whole embryonic intestines at E12.5 are shown in B. *Gapdh* was used as a library control.

The fact that the zinc finger transcription factor Nr2f1 and its paralogue Nr2f2 have both been previously implicated in the control of gliogenesis (Naka et al., 2008; Yamaguchi et al., 2004) combined with our analyses of both the transgene insertion site and the molecular signature of colonizing ENCCs (Figs 3 and 4) pointed to transgene-insertion-mediated derepression of *Nr2f1* as being responsible for the *Spot* ENS defects. To verify this model, we first evaluated whether the *Nr2f1-A830082K12Rik* gene pair normally interacts with the silencer element near the transgene insertion site using chromosome conformation capture (3C) assays. Using Neuro-2a cells, we found that the 5′ region shared by both *Nr2f1* and *A830082K12Rik* could specifically interact with the silencer element and not with other non-coding sequences located on the telomeric side (Fig. S9). Importantly, such a specific interaction was also detected using wild-type embryonic intestines but not using *Spot*^Tg/Tg^ tissues (Fig. 5A,B), strongly supporting the idea that the *Spot* transgenic insertion prevents an intra-chromosomal long-range interaction that normally attenuates *Nr2f1-A830082K12Rik* expression in ENCCs. To determine whether overexpression of *Nr2f1* alone is, at least in part, responsible for the colonic aganglionosis observed in *Spot*^Tg/Tg^ animals, we then generated E15.5 transgenic embryos that specifically co-expressed *Nr2f1* and *GFP* in NCCs under the control of a previously described *Sox10* enhancer (U3) (Antonellis et al., 2008; Werner et al., 2007). Despite intense efforts, this approach was, however, found to be ineffective. From a total of 350 recovered embryos, only two displayed GFP fluorescence and thus had their intestines dissected out. Of these dissected intestines, only one was of sufficient quality for subsequent analysis of the extent of ENCC colonization by performing immunofluorescence analysis of βIII-tubulin. Nevertheless, analysis of this single transgenic tissue revealed incomplete ENCC colonization of the colon (Fig. S10). Although no definitive conclusion can be drawn from a single sample, this encouraging result supports the hypothesis that *Nr2f1* overexpression can cause colonic aganglionosis independently of it flanking lncRNA *A830082K12Rik*.

## DISCUSSION

Characterization of the *Spot* mouse line suggests that mutation or disruption of *Nr2f1*-*A830082K12Rik* silencer elements might constitute a new genetic explanation for Waardenburg syndrome and/or HSCR, with premature gliogenesis of ENCCs being the pathogenic mechanism underlying the aganglionic megacolon phenotype. Another important finding made with the *Spot* model is that vestibular melanocytes appear to play a key role in the control of endolymph homeostasis, in a way similar to that but independent of cochlear melanocytes.

### Contribution of non-coding sequences to neurocristopathies

Although disruption of remote non-coding DNA elements involved in the regulation of key NCC genes is believed to be an important cause of neurocristopathies (Amiel et al., 2010), very few examples have been described in humans or animal models. Pertaining to Waardenburg syndrome and/or HSCR, *Spot* is only the fourth such mouse model to be described, after *Hry*, *TashT* and *Holstein* (Antonellis et al., 2006; Bergeron et al., 2015; Soret et al., 2015). Of note, all four models have in common a causative mutation involving a transgenic insertion. In the *Hry* model of Waardenburg syndrome type IV, *Sox10* expression is strongly downregulated owing to transgene-insertion-mediated deletion of a strong enhancer (Antonellis et al., 2006), a mechanism that has subsequently been reported to occur in humans as well (Bondurand et al., 2012; Lecerf et al., 2014). In contrast, for the other three recently described lines, the transgenic insertion disrupts a long-range interaction involved either in the repression [*Fam162b* in the *TashT* line (Bergeron et al., 2015) and *Nr2f1*-*A830082K12Rik* in the *Spot* line (this work)] or insulation [*Col6a4* in the *Holstein* line (Soret et al., 2015)] of the affected gene(s). In addition to these regulatory non-coding DNA elements, the *Spot* line now opens up the possibility that lncRNAs might be another type of non-coding sequence that could contribute to Waardenburg syndrome and/or HSCR. Indeed, even though our transient transgenic data suggest that *Nr2f1* overexpression is sufficient by itself to phenocopy the *Spot* ENS defect (i.e. colonic aganglionosis), we cannot rule out the possibility that the lncRNA *A830082K12Rik*, which is transcribed in the reverse orientation relative to *Nr2f1*, might play an upstream regulatory role in the *Spot* line. Additionally, given that *Nr2f1* and *A830082K12Rik* are both overexpressed in *Spot* ENCCs, it is tempting to speculate that *A830082K12Rik* could be involved in the transcriptional activation of its flanking gene. Such a cis-acting positive role on transcription has been described previously in a number of similar cases (Guil and Esteller, 2012; Quinn and Chang, 2015) and definitely represents an intriguing possibility to test in future work.

### A role for *Nr2f1* in enteric gliogenesis

Our study is the first to experimentally demonstrate *in vivo* that premature glial differentiation of ENCCs can represent a valid pathological mechanism for HSCR. Indeed, although this mechanism was first suggested from studies using mouse models with constitutive activation of Hedgehog signaling in ENCCs, whether the observed defects were sufficient to trigger megacolon could not be determined owing to embryonic lethality between E12.5 and E14.5 (Liu et al., 2015; Ngan et al., 2011). By contrast, it is interesting to note that the premature gliogenesis observed in these models has been reported to negatively impact the migration (Liu et al., 2015) and proliferation (Ngan et al., 2011) of ENCCs in a way similar to that which we observed in the *Spot* line. Taken together with our RNAseq data showing that the Hedgehog pathway is not activated in *Spot* ENCCs (the Hedgehog autoregulatory targets *Gli1* and *Ptch1* are even downregulated by 2.14- and 1.58-fold, respectively; Table S1), this raises the possibility that *Nr2f1* might lie downstream of Hedgehog signaling in the gene regulatory network that controls enteric gliogenesis. Moreover, a role for the known Hedgehog target *Nr2f2* (Krishnan et al., 1997; Takamoto et al., 2005) is also expected. Indeed, our RNAseq data indicate that *Nr2f1* and *Nr2f2* are both expressed to similar levels in wild-type ENCCs (Table S1), and their gene products (i.e. transcription factors of the orphan nuclear receptor superfamily) have been shown to be functionally redundant in the promotion of glial differentiation in the developing central nervous system (Naka et al., 2008). Such functional redundancy combined with the early death of *Nr2f1-* [at birth (Qiu et al., 1997)] and *Nr2f2-*null [at E12.5 (Pereira et al., 1999)] mutants most likely explains why these genes have not been previously associated with enteric gliogenesis. Determining the exact mechanism of action of Nr2f1 and Nr2f2 in enteric gliogenesis will require further work but, based on previous studies, it is tempting to speculate that they play both instructive [e.g. direct transcriptional regulation of pro-glial gene expression (Montemayor et al., 2010; Yamaguchi et al., 2004)] and permissive [e.g. allowing increased responsiveness to gliogenic cytokines (Naka et al., 2008)] roles.

### The role of vestibular melanocytes in the control of balance

Hearing loss is considered to be an important major feature of human Waardenburg syndrome (Pingault et al., 2010), but it was not observed in *Spot*^Tg/Tg^ animals using to the Preyer's reflex test at P21. Although it is possible that subtle hearing dysfunctions (e.g. unilateral and/or progressive hearing loss) might have been missed because of the nature of the test itself or the age at which it was performed, the fact that vestibular but not cochlear melanocytes were absent in *Spot*^Tg/Tg^ inner ears is strongly supportive of a vestibule-restricted phenotype. In this regard, it is interesting to note that circling behavior without evidence of hearing loss has been previously reported in the *Slugh*^−/−^ (*Snai2*^−/−^) mouse model of Waardenburg syndrome type 2 (Sanchez-Martin et al., 2002). Comparison to other mouse lines also indicates that the endolymphatic collapse observed in *Spot*^Tg/Tg^ vestibular sensory epithelia is similar to what has been described in the cochlea of *Mitf*^−/−^ and *Ednrb*^−/−^ models of Waardenburg syndrome type 1 and type 4, respectively (Koide et al., 1998; Matsushima et al., 2002; Tachibana et al., 1992). Endolymphatic collapse in these models has been attributed to dysregulation of the K^+^-high and Na^+^-low ionic composition of endolymph secondary to the loss of melanocytes in the stria vascularis compartment of the cochlea (Tachibana et al., 2003). Because these so-called intermediate cells are crucially required to transport K^+^ ions from the spiral ligament region towards the K^+^-secreting marginal cells that line the endolymph compartment (Zdebik et al., 2009), our data strongly indicate a similar role for vestibular melanocytes. In total accordance with this hypothesis, it is noteworthy that vestibular melanocytes are located in a similar manner underneath the K^+^-secreting dark cell layer that line the endolymphatic space of vestibular sensory organs (Forge and Wright, 2002). By contrast, the reason why *Spot*^Tg/Tg^ vestibular melanocytes are affected but cochlear melanocytes are not is currently unknown. In fact, this question might be better addressed by determining why cochlear melanocytes appear to be specifically resistant to a problem that leads to the depletion of other subpopulations of melanocytes (i.e. in the vestibule and skin). Determining whether premature gliogenesis and/or other pathological mechanisms are involved will represent other exciting lines of research to be performed with the *Spot* mouse line.

## MATERIALS AND METHODS

### Animals

Work with mice was performed in accordance with the guidelines of the Canadian Council on Animal Care (CCAC) and approved by the relevant institutional committee [Comité institutionnel de protection des animaux (CIPA); CIPA reference #650] of University of Quebec at Montreal (UQAM). Mouse were euthanized by using gradual-fill carbon dioxide (CO_2_) gas following isoflurane anesthesia. *Spot* transgenic mice and *Nr2f1*-overexpressing embryos were generated by using standard pronuclear microinjection (Nagy et al., 2003) into FVB/N and B6C3 embryos, respectively.

For the *Spot* line, two transgenes were co-injected at equimolar ratio: a tyrosinase minigene (Fig. S11) that allows visual identification of transgenic animals through rescue of pigmentation (Methot et al., 1995) and a GFP-tagged βarrestin-2 (GFP-βarr2) construct (not expressed in the *Spot* line). To endogenously label NCCs with fluorescence, *Spot* animals were intercrossed with previously described *Gata4*p[5 kb]-GFP (G4-GFP) or -RFP (G4-RFP) mice, also maintained on a FVB/N background (Pilon et al., 2008). To generate embryos, mice were mated overnight, and noon on the day of vaginal plug detection was designated as E0.5.

For *Nr2f1* transient transgenics, the *Nr2f1* open reading frame was subcloned from a vector kindly provided by Ming-Jer Tsai (Baylor College of Medicine, Houston, Texas) into a previously described plasmid (Bergeron et al., 2015) that allows co-expression with GFP under the control of the *Hsp68* minimal promoter (Kothary et al., 1989) and a NCC-specific *Sox10* enhancer (U3, also known as MCS4) (Antonellis et al., 2008; Werner et al., 2007). Embryos were collected 15 days after microinjection and individually analyzed for GFP fluorescence. Whole intestines of GFP-expressing embryos were then labeled with anti-βIII-Tubulin antibodies (see below).

### Labeling and imaging of embryonic and postnatal tissues

Labeling of embryonic and postnatal tissues by using immunohistochemistry, immunofluorescence, staining of acetylcholinesterase activity or a TUNEL assay (*in situ* cell death detection kit, TMR red; Roche Applied Science) was performed as previously described (Bergeron et al., 2015; Sanchez-Ferras et al., 2016; Soret et al., 2015). All antibodies used in this study (including dilution factors) are listed in Table S2.

For inner ear analyses, temporal bones were harvested and fixed in 4% paraformaldehyde overnight at 4°C. For intracranial imaging of melanocytes, bones were simply cleared in methylsalicylate. For membranous labyrinth analyses with DAPI (5 µg/ml; Sigma) and TRITC–Phalloidin (50 µg/ml; Sigma) fluorescent staining, bones were demineralized (using several overnight washes of 0.5 M EDTA in PBS) and vibratome sectioned (100 µm for P1 tissues or 200 µm for P20 tissues). For microcomputed tomography analysis, bones were scanned with a Skyscan 1172c X-ray system (Soquelec, Canada) using the following settings: 5-μm image pixel size, 80 kV, 100 μA and 900-ms exposure time through a 0.5 mm Al filter. Raw images were reconstructed with the NRecon software (Skyscan, Belgium); bony labyrinth volumes of interest were defined, modeled and analyzed with CTAn software (Skyscan, Belgium).

Pigmented melanocytes as well as endogenous GFP- (in *Spot*;G4-GFP tissues) or RFP-labeled cells (in *Spot*;G4-RFP tissues) were visualized using a Leica M205FA fluorescence stereomicroscope, whereas immunohistochemistry slides were observed using a DM2000 Leica upright microscope. In each case, images were acquired with a Leica DFC495 digital camera and the Leica Application Suite (LAS) software (Leica microsystems). Fluorescent cells and tissues were also examined using an inverted Nikon TI microscope and imaged with a Nikon A1 confocal unit and the NIS-Element AR4 software (Nikon). All images were processed using the ImageJ software.

### Fluorescence-activated cell sorting

Whole intestines at relevant stages (E12.5 or E13.5) were obtained from *Spot*^Tg/+^;G4-RFP intercrosses and individually processed for dissociation at 37°C in Eagle's minimal essential medium (EMEM) containing collagenase (0.4 mg/ml; Sigma C2674), dispase II (1.3 mg/ml; Life Technologies 17105-041) and DNAse I (0.5 mg/ml; Sigma DN25). A MoFlo XDP (Beckman Coulter) cell sorter was then used to collect single RFP-positive viable cells from each dissociated intestinal tissue (∼15,000 cells per intestine on average). Samples of FACS-recovered cells from individual embryos were subsequently kept at −80°C, and the genotype of each sample was determined by performing PCR using the corresponding embryo head as DNA source (see below). For RNA extraction, RFP-positive cells from single embryos were processed for semi-quantitative RT-PCR analyses, whereas RFP-positive cells from groups of 5-6 embryos were processed for RNAseq studies. Total RNA was extracted using the RNeasy Plus purification mini kit in accordance with the manufacturer's instructions (Qiagen).

### Genotyping PCR and semi-quantitative RT-PCR

Genotyping of the *Spot* allele was performed using PCR with a standard Taq DNA polymerase (Feldan) and primers flanking the insertion and deletion sites. For semi-quantitative RT-PCR, 10 ng of total RNA was reverse-transcribed using the Superscript II enzyme (Life Technologies, Canada) and a poly-dT_17_ primer in accordance with manufacturer's instructions. 1 µl of the resulting cDNA pool was then used for amplification of the desired target using specific primers. The expression level of the housekeeping gene *Gapdh* was used for normalization. PCR consisted of 35 cycles of: 30 s at 95°C, 40 s at 60°C and 30 s at 72°C. Amplicons were resolved on a 2% agarose gel. Primer details can be found in Table S3.

### High-throughput genome and transcriptome sequencing

Whole genome and transcriptome library generation and sequencing (HiSeq 2000 platform, Illumina) as well as bioinformatics analyses were performed by McGill University and Génome Québec Innovation Centre as previously described (Bergeron et al., 2015). For RNA sequencing, 100 ng of total RNA was used to prepare rRNA-depleted libraries, and three biological replicates per genotype were analyzed (each containing FACS-recovered ENCCs from 5-6 embryos). From 300-500-bp library inserts, ∼200 million paired-end sequences (100 bp length) were obtained for the *Spot*^Tg/Tg^ genomic DNA (i.e. ∼30× coverage), whereas ∼60 million paired-end sequences were obtained for each of the transcriptome samples. All sequences were mapped onto the mm10 *Mus musculus* reference genome. Differential gene expression data were generated using DESeq and edgeR R Bioconductor packages with *P*-values adjusted using the false discovery rate (Benjamini–Hochberg) procedure. For the GO analysis, only differentially-expressed genes with an adjusted edgeR *P*-value ≤0.001 were included. The GO analysis was performed using GOToolBox (http://genome.crg.es/GOToolBox) with enriched categories selected from a hypergeometric test based on a Benjamini–Hochberg-corrected *P*-value threshold of 0.05. REVIGO (http://revigo.irb.hr) was used to generate a treemap that integrates all enriched and non-redundant GO terms in higher level categories (Supek et al., 2011). Fragments per kilobase of transcript per million mapped reads (FPKM) analysis was performed and normalized with the Cufflinks program.

### *Ex vivo* time-lapse imaging of ENCCs

Live imaging of ENCCs was performed using a previously described suspended culture technique (Nishiyama et al., 2012). Briefly, E12.5 and E13.5 whole intestines from control (G4-RFP) and mutant (*Spot*^Tg/Tg^;G4-RFP) embryos were placed on a small nitrocellulose filter (Millipore GSWP01300), which was then flipped on top of paraffin lines streaked in parallel on a 60-mm cell culture dish (Corning). Samples were cultured in Dulbecco's modified Eagle's medium (DMEM)/F12 medium containing 10% FBS and penicillin-streptomycin for 6 h (at 37°C and 5% CO_2_) in a microscope incubation chamber (Okolab), and 250-μm-thick stacks were acquired every 10 min using a Nikon A1R confocal unit and a 10× objective. For calculations of speed and directionality of cell migration, 6-7 chain tip cells were tracked from at least three intestines of each genotype as previously described (Bergeron et al., 2015; Soret et al., 2015). The net cell trajectory was determined by drawing a straight line relative to the longitudinal axis of the intestinal tissue from the cell position at 0 h and the radial position (in degrees) of the same cell at 6 h.

### Luciferase reporter and chromosome conformation capture assays

Constrained elements around the *Spot* insertion site were identified using the 60 vertebrate basewise conservation track of the UCSC genome browser (genome.ucsc.edu). A 676-bp highly conserved sequence telomeric to the insertion site was PCR-amplified (oligonucleotides sequences can be found in Table S3), cloned into the pGEM-T vector (Promega) and validated by sequencing. A luciferase reporter construct was generated by subcloning the constrained element into a modified pXP2 vector containing the 109-bp minimal promoter of thymidine kinase (Nordeen, 1988). Maintenance and transfection of P19 embryocarcinoma and Neuro-2a neuroblastoma cells (Sanchez-Ferras et al., 2014), as well as 3C assays (oligonucleotides sequences can be found in Table S3) in cells and embryonic guts (Bergeron et al., 2015), were performed as per our previous work.

### Statistics

Data are presented as mean±s.d., with the number of measurements (*n*) and the number of animals used (*N*) included in the figure and/or legend where appropriate. Statistical significance of quantified data was determined using the Student's *t*-test (two-tailed) or the likelihood ratio test (for directionality data). Differences were considered statistically significant when the *P*-value was less than 0.05.
